# Supplementary material for: Characterization of GafChromic EBT2 film dose measurements using a tissue-equivalent water phantom for a Theratron® Equinox Cobalt-60 teletherapy machine
Source: PLoS One. 2022 Aug 19;17(8):e0271000. doi: 10.1371/journal.pone.0271000 (PMC9390906; doi:10.1371/journal.pone.0271000)

APPENDIX A

AutoCAD Design of encapsulation cap Option One

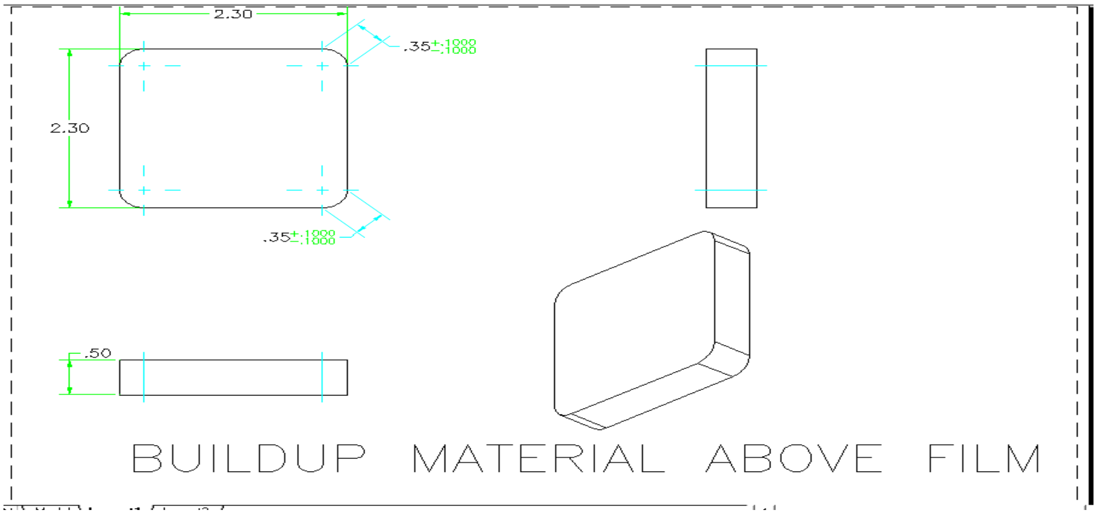

Figure A1: 2D dimension of buildup material above film

** All measurements are in centimeters*

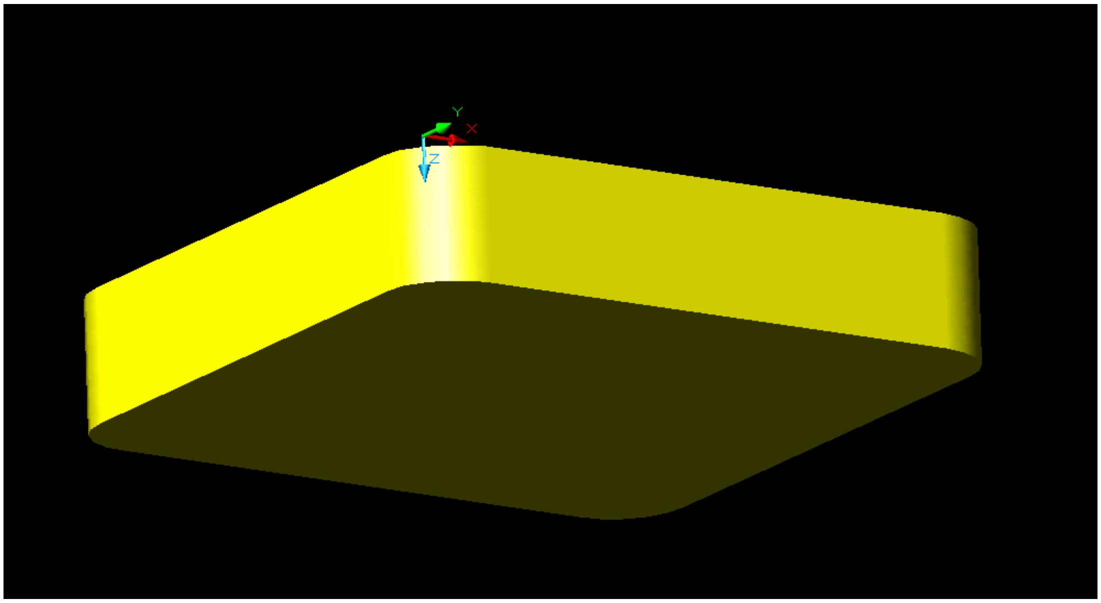

Figure A2: Buildup cap above film

University of Ghana http://ugspace.ug.edu.gh

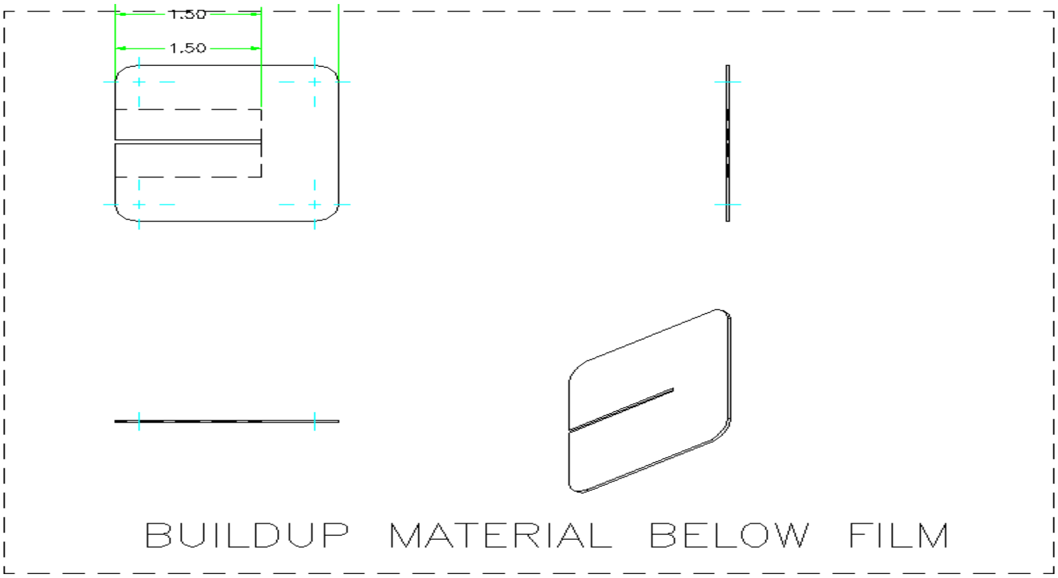

Figure A3: 2D dimension of buildup cap below film

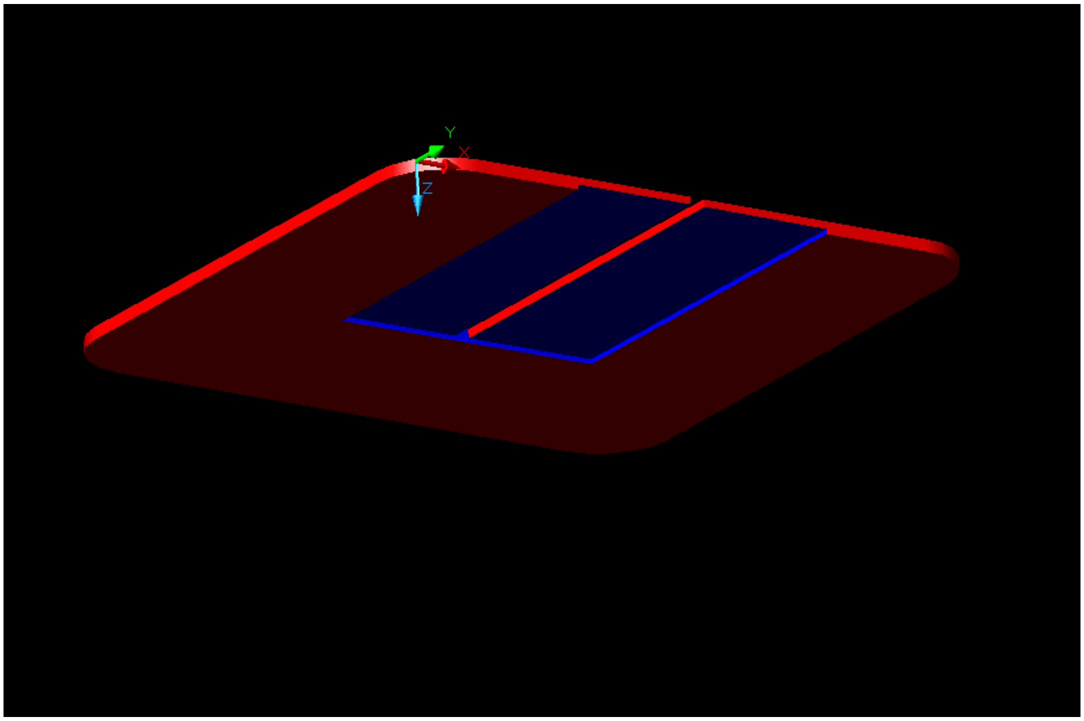

Figure A4: 3D buildup cap below film

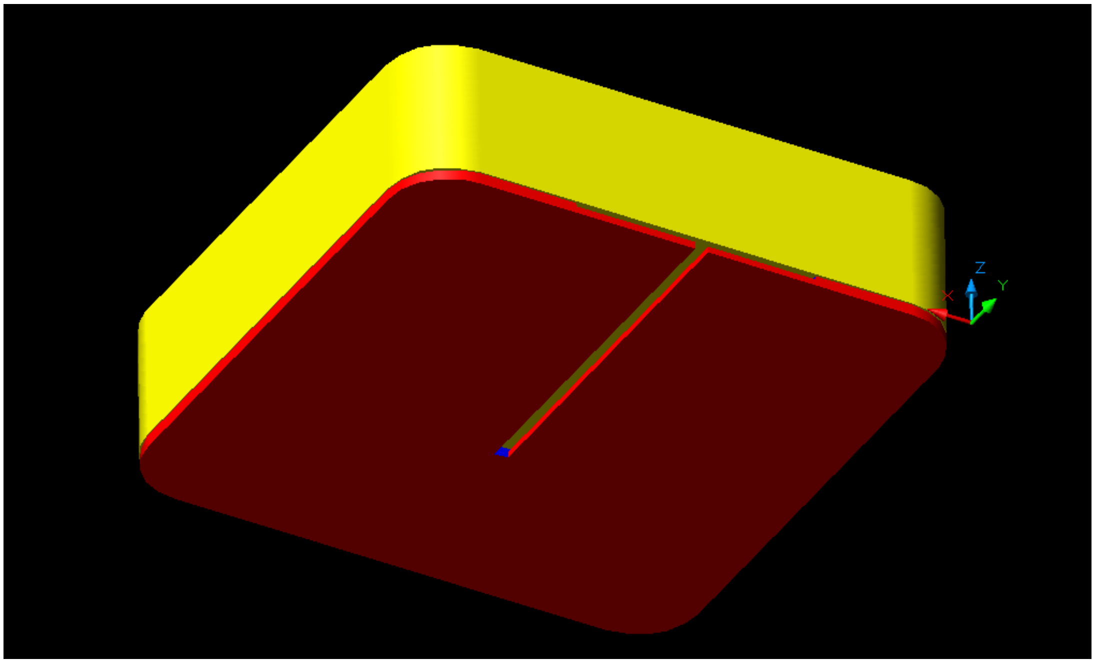

Figure A5: 3D assembly drawing of buildup cap with film slot

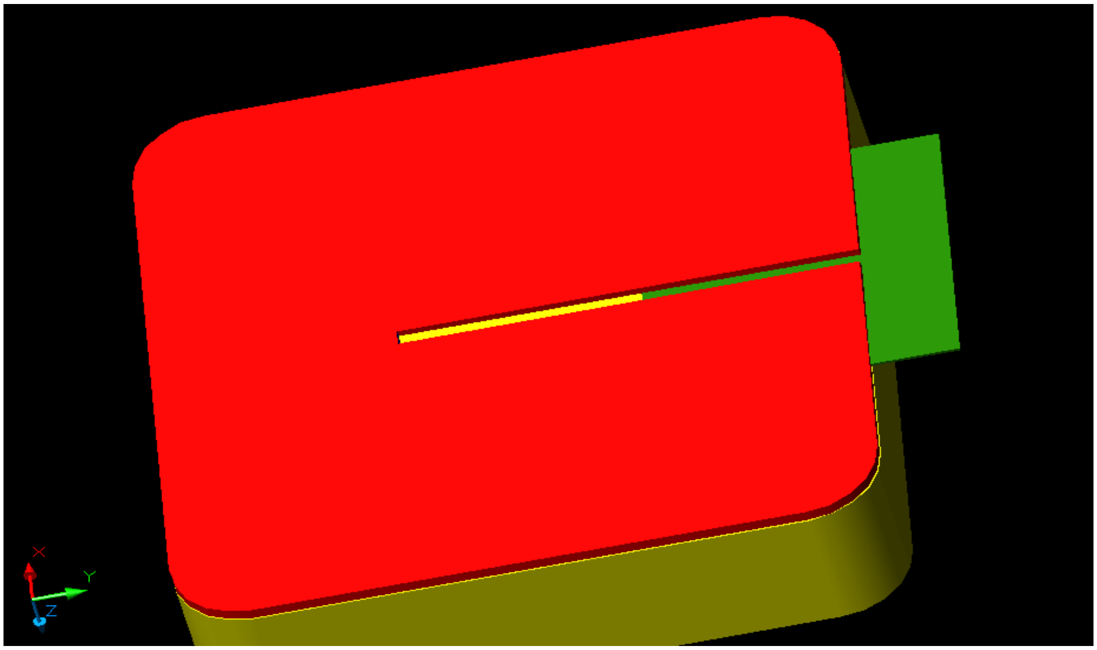

Figure A6: 3D assembly drawing of buildup cap with film being inserted

University of Ghana http://ugspace.ug.edu.gh

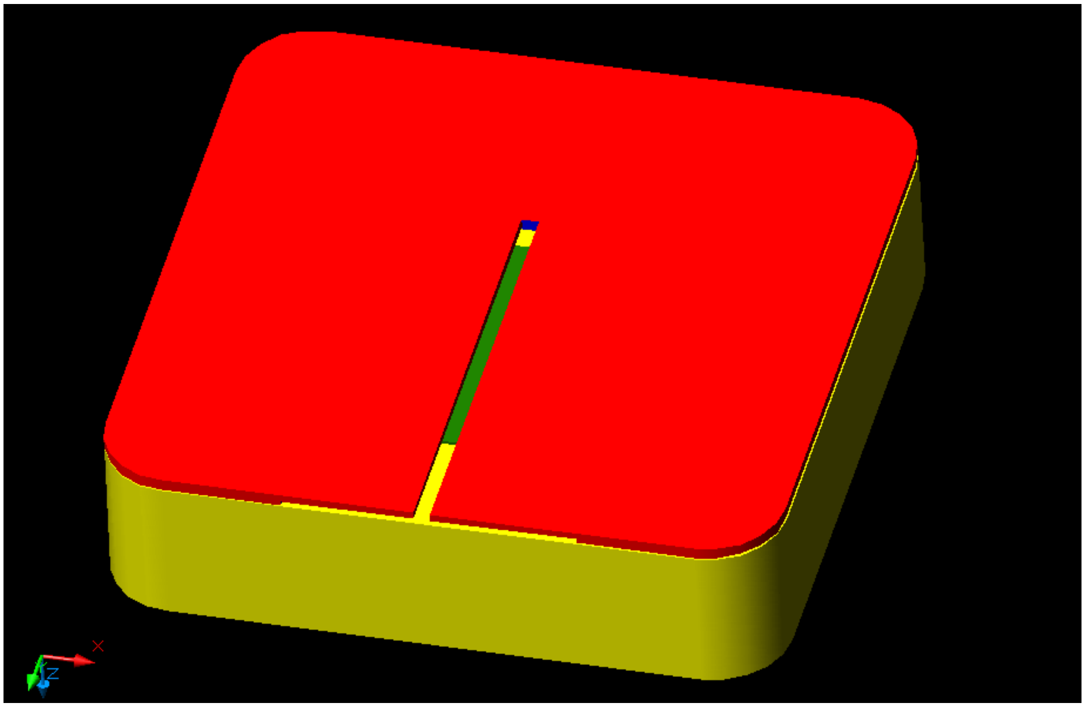

Figure A8: 3D assembly drawing of fully inserted film in buildup cap

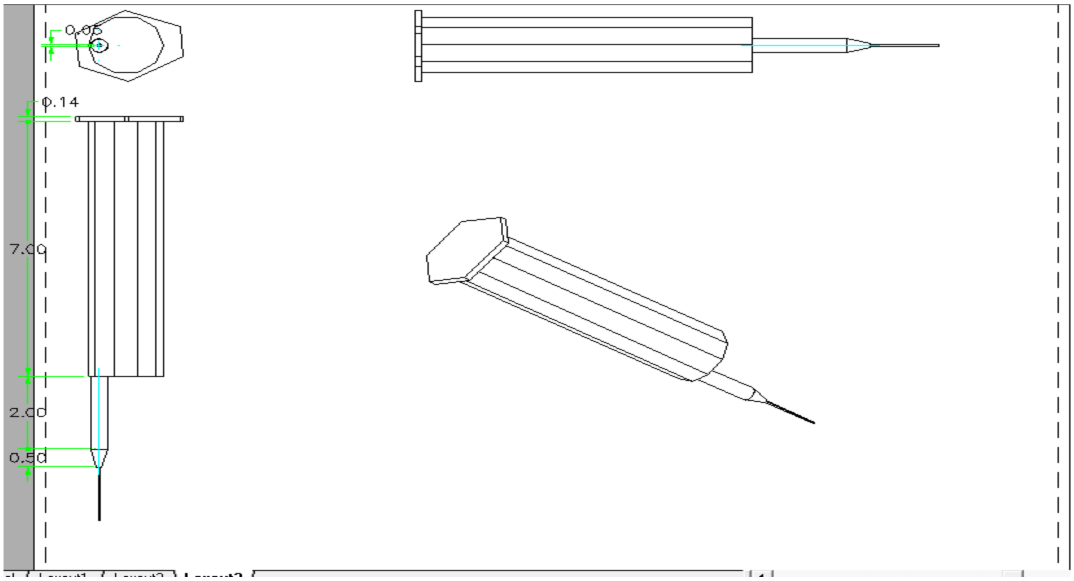

Figure A9: 2D dimension of film handler

***This is a device designed to aid the insertion and removal of the film from the cap.**

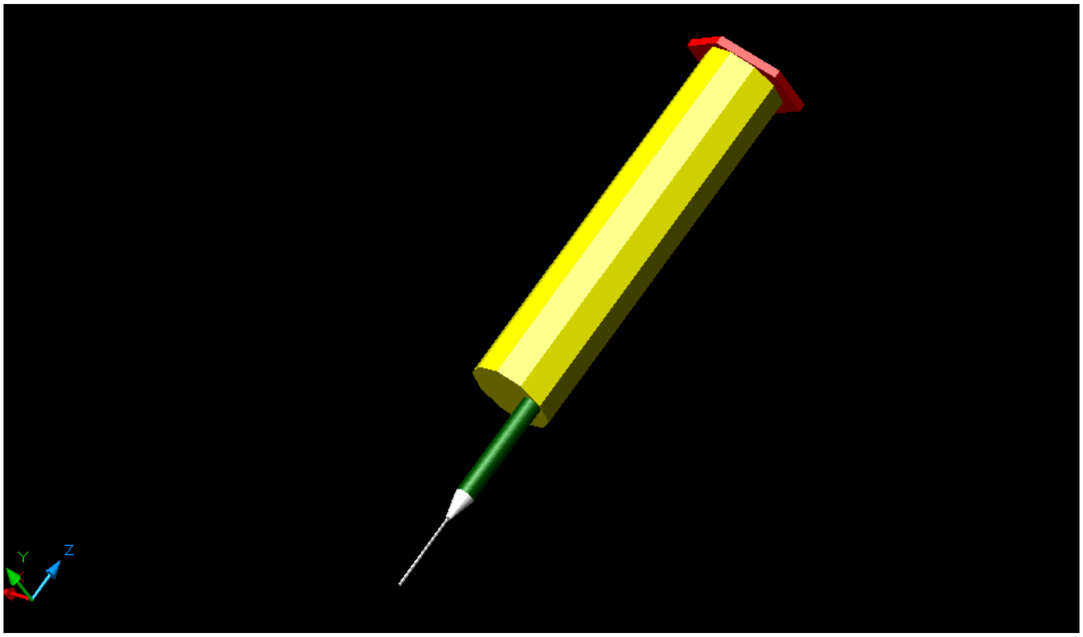

Figure A10: 3D assembly drawing of film handler

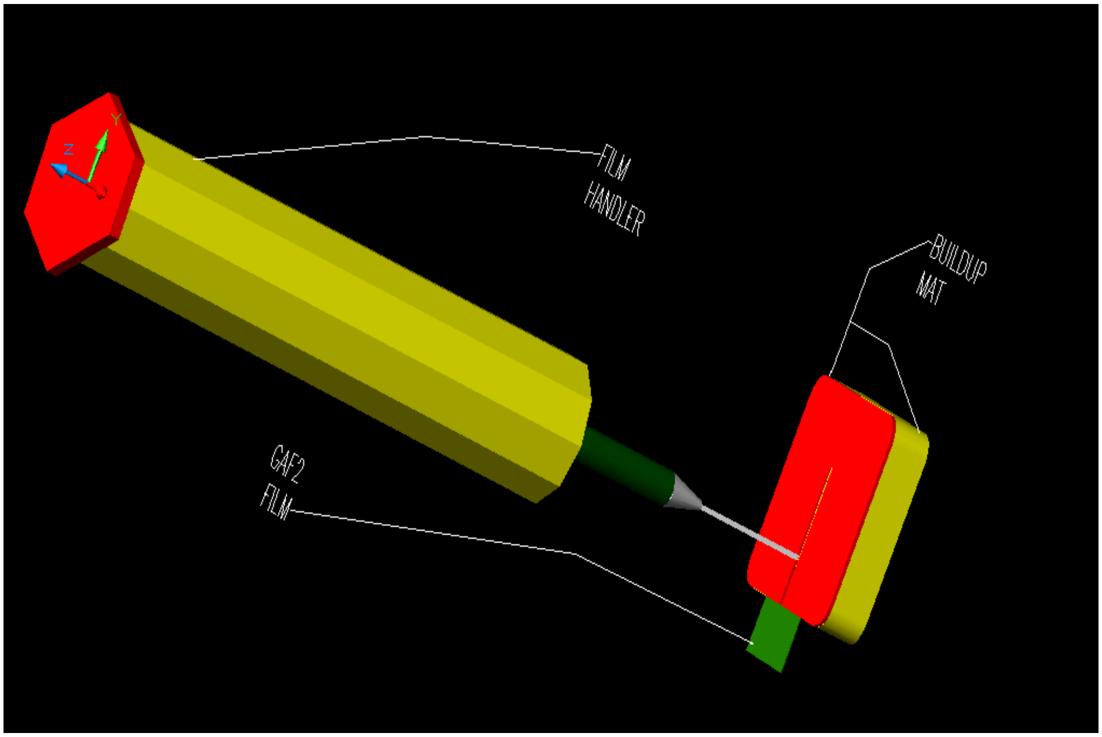

Figure A11: 3D assembly drawing showing film being pulled with film handler

University of Ghana http://ugspace.ug.edu.gh

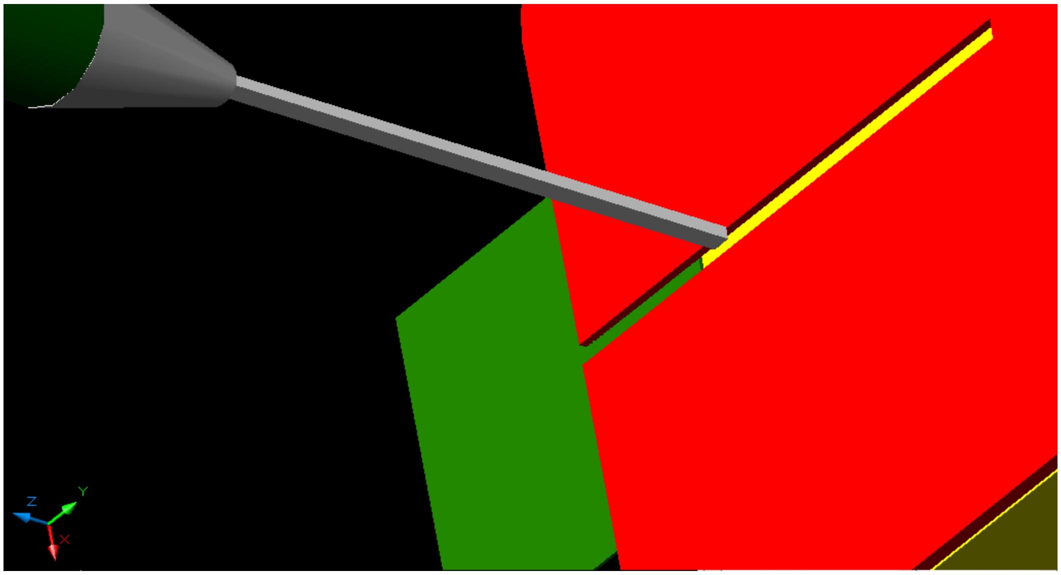

Figure A12: Closer view of film handler, film, buildup cap assembly

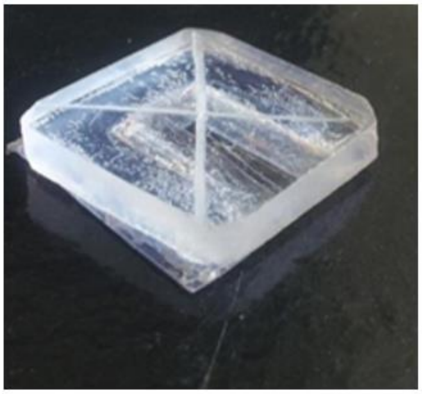

Figure A13: Physically constructed Option One

APPENDIX B

Design of encapsulation cap OPTION TWO

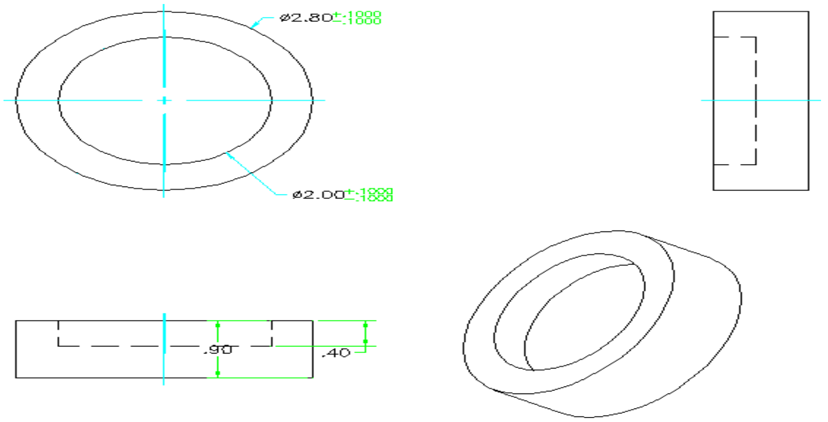

Figure B1: 2D dimension of buildup cap

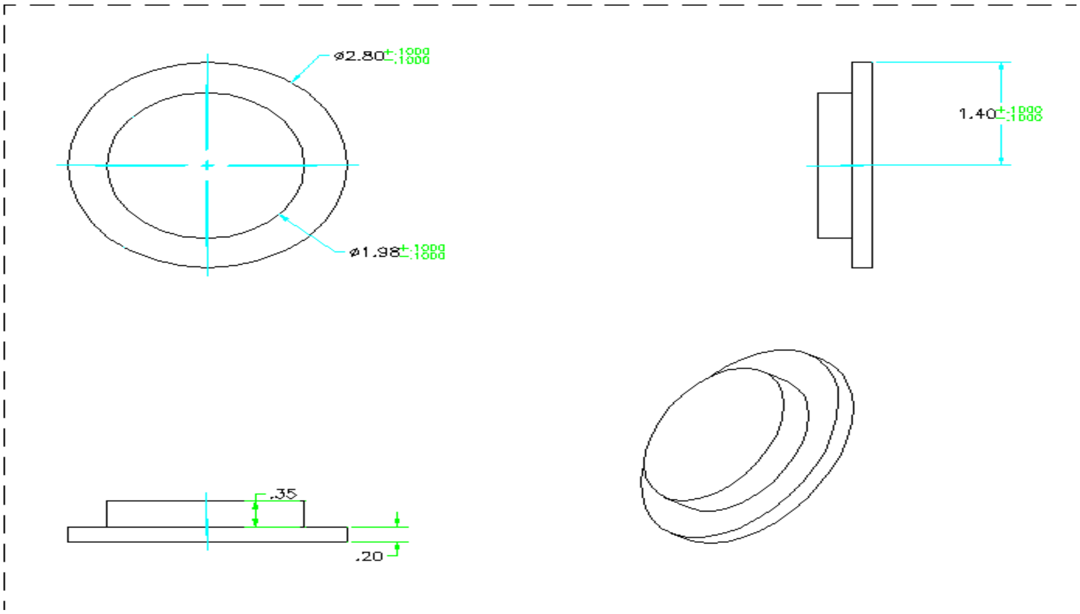

Figure B2: 2D dimension of buildup cap locker

University of Ghana http://ugspace.ug.edu.gh

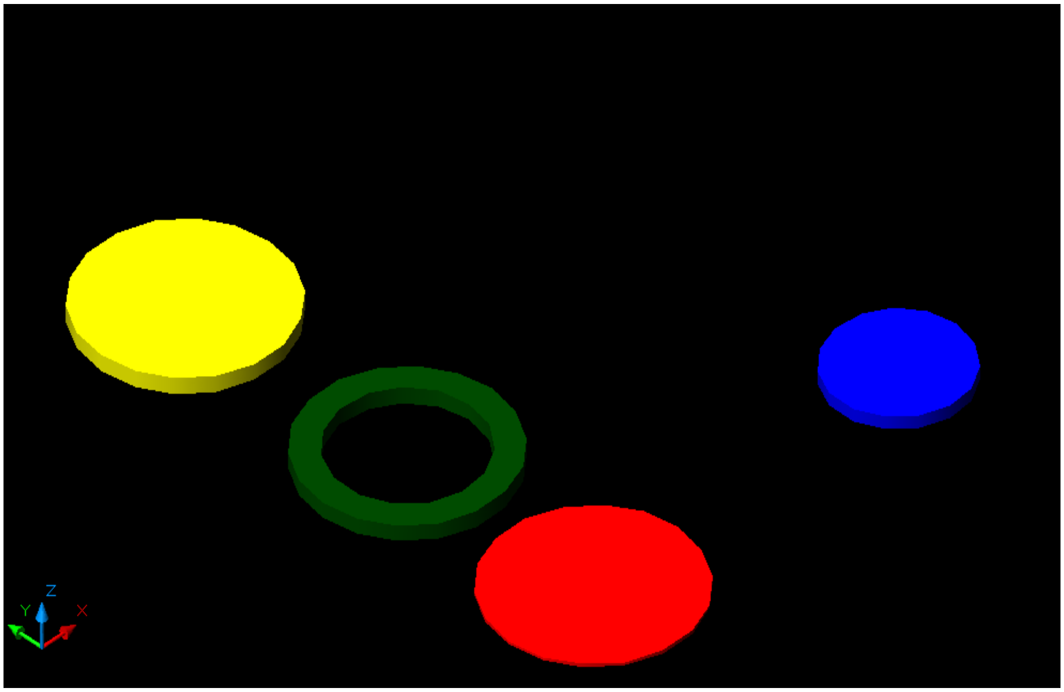

Figure B3: 3D Part design of buildup cap assembly

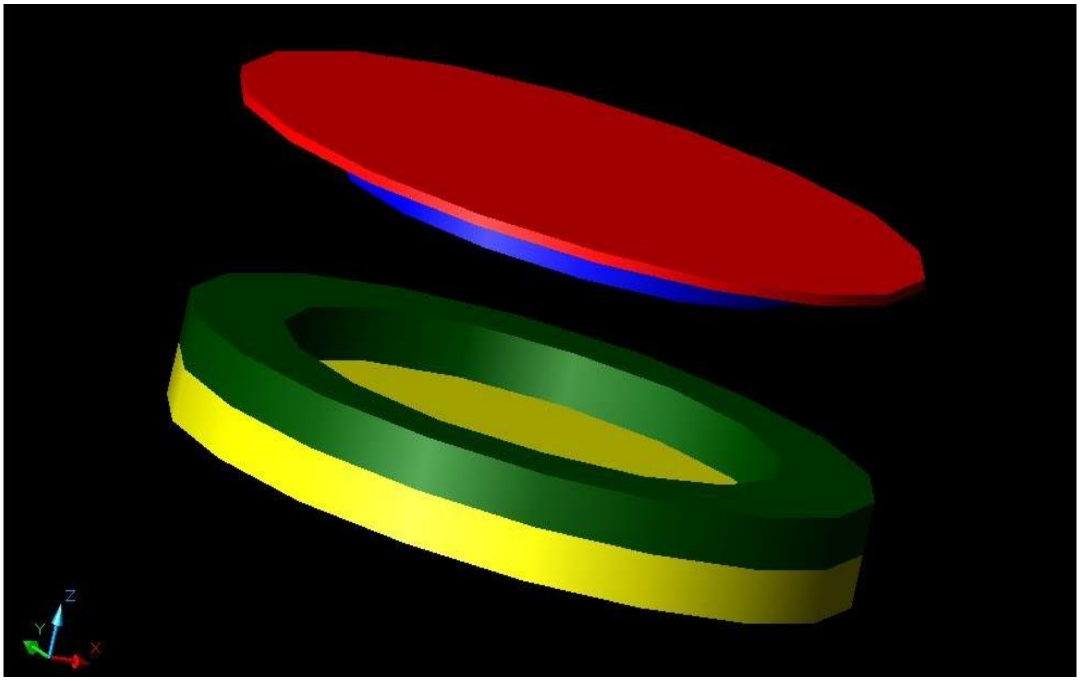

Figure B6: 3D Assembly drawing showing locker in position

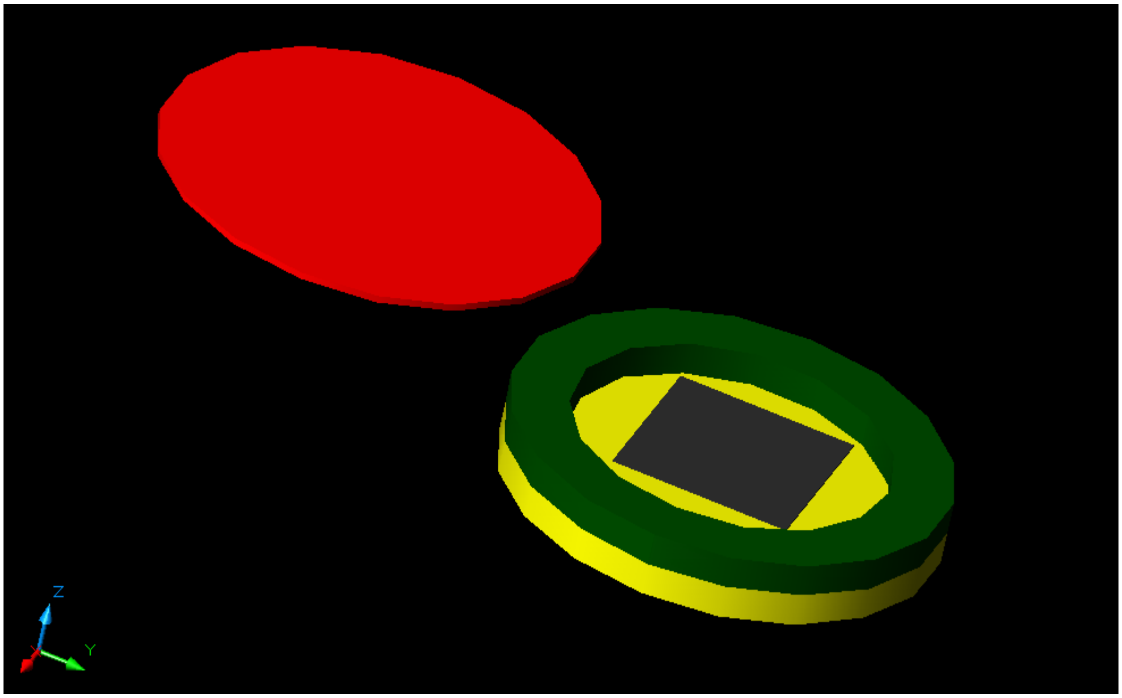

Figure B5: 3D Assembly drawing showing GAF2 in position

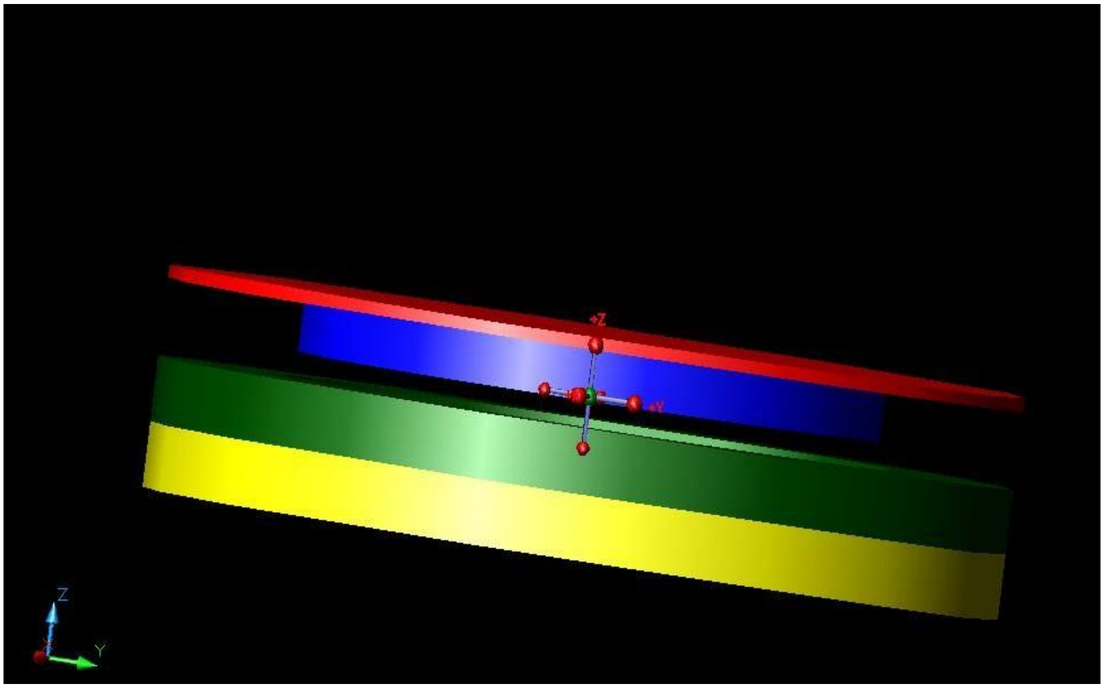

Figure B6: 3D Assembly drawing showing locker in position

University of Ghana http://ugspace.ug.edu.gh

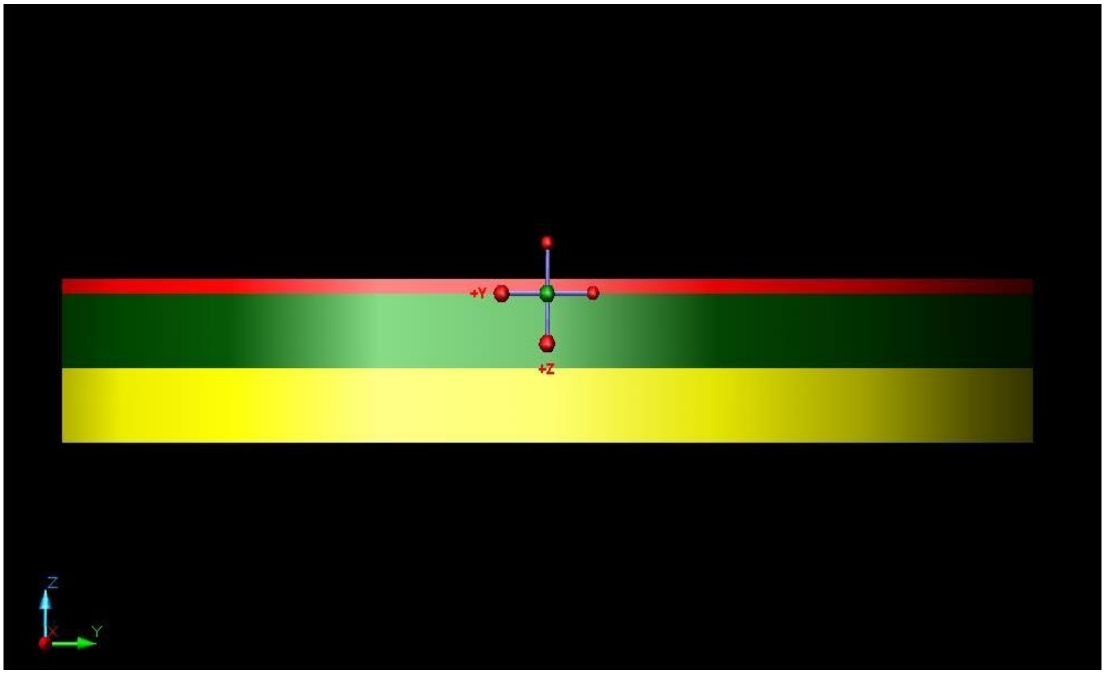

Figure B7: 3D Assembly drawing showing fully encapsulated GAF2

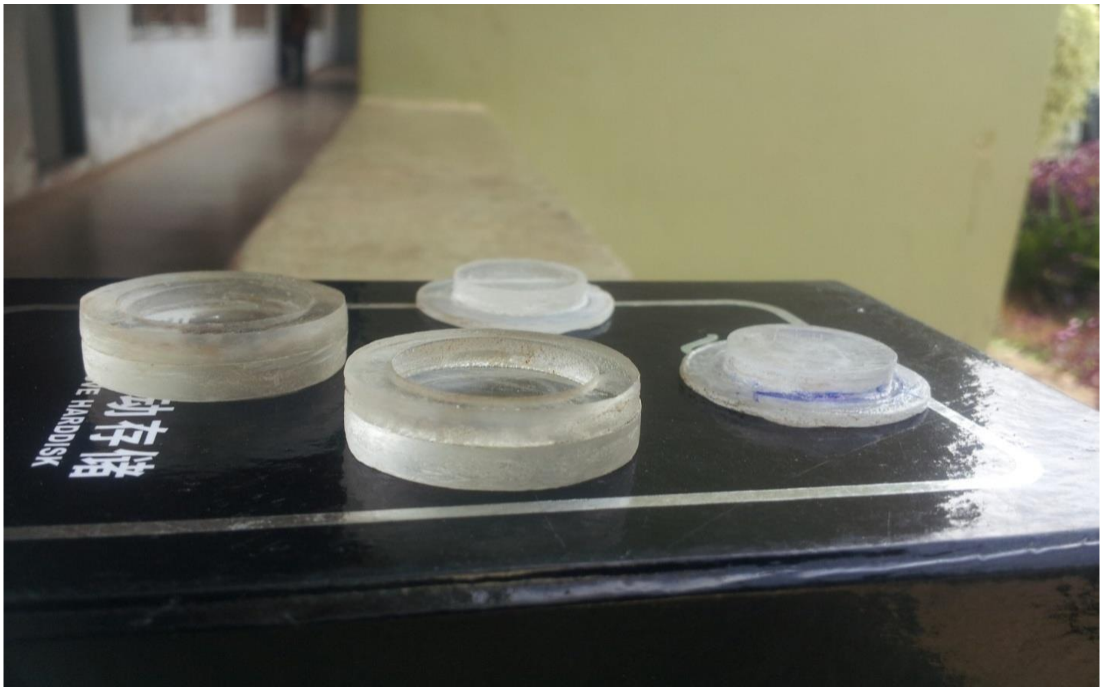

Figure B8: Physically constructed Option Two

University of Ghana http://ugspace.ug.edu.gh

APPENDIX C

Post-exposure optical density growth

Table C1: Post-exposure mean optical density growth for different doses of radiation

Time after exposure (minutes)	50 cGy	200 cGy	400 cGy	800 cGy
1	0.02	0.14	0.28	0.48
60	0.03	0.15	0.29	0.49
120	0.03	0.15	0.30	0.49
180	0.03	0.15	0.30	0.49
240	0.03	0.15	0.30	0.50
300	0.03	0.15	0.30	0.50
360	0.04	0.15	0.32	0.50
420	0.04	0.15	0.32	0.50
480	0.05	0.16	0.32	0.50
540	0.05	0.16	0.33	0.50
600	0.05	0.16	0.33	0.51
660	0.05	0.16	0.34	0.51
720	0.05	0.17	0.34	0.51
780	0.05	0.17	0.34	0.53
840	0.05	0.17	0.34	0.53
900	0.05	0.17	0.34	0.53
960	0.05	0.17	0.34	0.53

University of Ghana http://ugspace.ug.edu.gh

Table C2: Post-exposure optical density growth for different doses of radiation

Time after exposure (minutes)	50 cGy	200 cGy	400 cGy	800 cGy
1020	0.05	0.19	0.34	0.53
1080	0.05	0.19	0.35	0.54
1140	0.05	0.19	0.35	0.54
1200	0.05	0.19	0.35	0.54
1260	0.05	0.19	0.35	0.55
1320	0.05	0.19	0.35	0.55
1380	0.05	0.19	0.35	0.55
1440	0.05	0.19	0.35	0.55
2160	0.05	0.19	0.35	0.55
2880	0.05	0.19	0.35	0.56
3600	0.05	0.19	0.35	0.56
4320	0.05	0.19	0.35	0.56
5760	0.05	0.19	0.35	0.56
6480	0.05	0.19	0.35	0.56

University of Ghana http://ugspace.ug.edu.gh

APPENDIX D

Calibration curve

Table D1: Relationship between absorbed dose to GAF2 and net optical density of GAF2

Dose (cGy)	R1	R2	R3	MEAN	SD
0	0.00	0.00	0.00	0.00	0.00
50	0.05	0.04	0.06	0.05	0.01
100	0.11	0.11	0.11	0.11	0.00
150	0.16	0.15	0.17	0.16	0.01
200	0.18	0.20	0.19	0.19	0.01
250	0.23	0.22	0.24	0.23	0.01
300	0.28	0.28	0.28	0.28	0.00
350	0.30	0.31	0.32	0.31	0.01
400	0.35	0.35	0.35	0.35	0.00
450	0.39	0.38	0.40	0.39	0.01
800	0.56	0.57	0.55	0.56	0.01

Note:

* R1, R2, R3 stands for reading 1, reading 2 and reading 3 respectively

* SD stands for standard deviation

University of Ghana http://ugspace.ug.edu.gh

APPENDIX E

EFFECT OF FIELD SIZE ON GAF2 RESPONSE

Table E1: Effect of field sizes on bare film optical density

FIELD SIZE (cm^2)	R1	R2	R3	MEAN	SD	ND
4×4	0.03	0.03	0.02	0.03	0.01	0.67
6×6	0.03	0.02	0.03	0.03	0.01	0.67
8×8	0.04	0.03	0.04	0.04	0.01	0.91
10×10	0.04	0.04	0.04	0.04	0.00	1.00
12×12	0.05	0.06	0.05	0.05	0.01	1.33
14×14	0.05	0.06	0.05	0.05	0.01	1.33
16×16	0.06	0.05	0.06	0.06	0.01	1.42
18×18	0.08	0.08	0.08	0.08	0.00	2.00
20×20	0.08	0.08	0.08	0.08	0.00	2.00
22×22	0.09	0.07	0.08	0.08	0.01	2.00
24×24	0.08	0.08	0.08	0.08	0.00	2.00
26×26	0.09	0.09	0.09	0.09	0.00	2.25

.

*Where: ND represents normalized data; SD represents standard deviation; R1, R2 & R3 represents GAF2 NOD reading.

University of Ghana http://ugspace.ug.edu.gh

EFFECT OF FIELD SIZE ON GAF2 RESPONSE

Table E2: Effect of field sizes on encapsulated GAF2 optical density

FIELD SIZE (cm^2)	R1	R2	R3	MEAN	SD	ND
4×4	0.15	0.15	0.15	0.15	0.00	0.88
6×6	0.17	0.17	0.17	0.17	0.00	1.00
8×8	0.17	0.17	0.17	0.17	0.00	1.00
10×10	0.17	0.17	0.17	0.17	0.00	1.00
12×12	0.17	0.17	0.18	0.17	0.01	1.02
14×14	0.18	0.17	0.18	0.18	0.01	1.04
16×16	0.18	0.18	0.18	0.18	0.00	1.06
18×18	0.18	0.18	0.18	0.18	0.00	1.06
20×20	0.18	0.18	0.18	0.18	0.00	1.06
22×22	0.18	0.18	0.18	0.18	0.00	1.06
24×24	0.18	0.18	0.18	0.18	0.00	1.06
26×26	0.18	0.18	0.18	0.18	0.00	1.06

*Where: ND represents normalized data; SD represents standard deviation; R1, R2 & R3 represents GAF2 NOD reading.

University of Ghana http://ugspace.ug.edu.gh

APPENDIX F

EFFECT OF SSD ON GAF2 RESPONSE

Table F1: Effect of SSD on encapsulated GAF2 optical density

SSD (cm)	R1	R2	R3	MEAN	SD	ND
70	0.38	0.39	0.38	0.38	0.01	2.40
75	0.36	0.37	0.36	0.36	0.01	2.27
80	0.26	0.28	0.27	0.27	0.01	1.69
85	0.23	0.22	0.22	0.22	0.01	1.10
90	0.20	0.20	0.20	0.20	0.00	1.25
95	0.19	0.19	0.19	0.19	0.00	1.19
100	0.16	0.16	0.16	0.16	0.00	1.00
105	0.16	0.15	0.15	0.15	0.01	0.96
110	0.13	0.13	0.13	0.13	0.00	0.81
115	0.12	0.12	0.12	0.12	0.00	0.75
120	0.12	0.11	0.12	0.12	0.01	0.73

*Where: ND represents normalized data;

SD represents standard deviation; R1, R2 & R3 represents GAF2 NOD reading.

University of Ghana http://ugspace.ug.edu.gh

EFFECT OF SSD ON GAF2 RESPONSE

Table F2: Effect of SSD on bare GAF2 optical density

SSD (cm)	R1	R2	R3	MEAN	SD	ND
70	0.07	0.07	0.07	0.07	0.00	1.50
75	0.06	0.07	0.06	0.06	0.01	1.36
80	0.05	0.06	0.06	0.06	0.01	1.21
85	0.05	0.05	0.07	0.06	0.01	1.21
90	0.07	0.06	0.06	0.06	0.01	1.36
95	0.06	0.05	0.05	0.05	0.01	1.14
100	0.05	0.04	0.05	0.05	0.01	1.00
105	0.04	0.03	0.04	0.04	0.01	0.79
110	0.02	0.03	0.03	0.03	0.01	0.57
115	0.02	0.02	0.02	0.02	0.00	0.43
120	0.01	0.02	0.01	0.01	0.01	0.29

*As shown in table 4.3 and table 4.4, for the same irradiation parameters, the mean optical density for encapsulated film was higher than that for bare film because the film in the encapsulated film is at depth of maximum dose, d_{max}. The maximum standard deviation per irradiation field for both encapsulated and bare film was observed to be 0.01. ND represents normalized mean optical density using reference SSD of 100 cm.

University of Ghana http://ugspace.ug.edu.gh

APPENDIX G

EFFECT OF GANTRY ANGLE ON GAF2 RESPONSE

Table G1: Effect of gantry angle on bare GAF2 optical density

GA (˚)	R1	R2	R2	MEAN	SD	ND
0	0.04	0.04	0.04	0.04	0.00	1.00
5	0.04	0.04	0.05	0.04	0.01	1.08
10	0.02	0.02	0.02	0.02	0.00	0.50
15	0.04	0.04	0.05	0.04	0.01	1.08
20	0.04	0.04	0.05	0.04	0.01	1.08
25	0.04	0.04	0.04	0.04	0.00	1.00
30	0.06	0.05	0.06	0.06	0.01	1.42
40	0.04	0.04	0.05	0.04	0.01	1.08
50	0.04	0.04	0.05	0.04	0.01	1.08
60	0.06	0.06	0.06	0.06	0.00	1.50
70	0.09	0.09	0.10	0.09	0.01	2.33
80	0.10	0.10	0.09	0.11	0.01	2.42
90	0.06	0.06	0.07	0.06	0.01	1.58
-5	0.05	0.04	0.05	0.05	0.01	1.17
-10	0.02	0.03	0.03	0.03	0.01	0.67
-15	0.04	0.04	0.05	0.04	0.01	1.08

University of Ghana http://ugspace.ug.edu.gh

-20	0.04	0.04	0.05	0.04	0.01	1.08
-25	0.04	0.05	0.05	0.05	0.01	1.17
-30	0.06	0.05	0.06	0.07	0.01	1.42
-40	0.04	0.04	0.05	0.04	0.01	1.08
-50	0.04	0.04	0.05	0.04	0.01	1.08
-60	0.06	0.06	0.06	0.06	0.00	1.50
-70	0.09	0.10	0.09	0.09	0.01	2.33
-80	0.10	0.10	0.10	0.10	0.00	2.50
-90	0.06	0.07	0.06	0.06	0.00	1.58

EFFECT OF GANTRY ANGLE ON GAF2 RESPONSE

Table G2: Effect of gantry angle on encapsulated GAF2 optical density

GA (˚)	R1	R2	R3	MEAN	SD	ND
0	0.16	0.16	0.16	0.16	0.00	1.00
5	0.16	0.16	0.16	0.16	0.00	1.00
10	0.16	0.16	0.16	0.16	0.00	1.00
15	0.16	0.16	0.16	0.16	0.00	1.00
20	0.17	0.17	0.17	0.17	0.00	1.00
25	0.16	0.16	0.16	0.16	0.00	1.00
30	0.16	0.16	0.16	0.16	0.00	1.00
40	0.16	0.16	0.16	0.16	0.00	1.00

University of Ghana http://ugspace.ug.edu.gh

50	0.16	0.16	0.16	0.16	0.00	1.00
60	0.16	0.16	0.16	0.16	0.00	1.00
70	0.17	0.16	0.17	0.17	0.01	1.00
80	0.16	0.16	0.16	0.16	0.00	1.00
90	0.16	0.16	0.16	0.16	0.00	1.00
-5	0.16	0.16	0.16	0.16	0.00	1.00
-10	0.16	0.16	0.16	0.16	0.00	1.00
-15	0.16	0.16	0.16	0.16	0.00	1.00
-20	0.16	0.16	0.16	0.16	0.00	1.00
-25	0.16	0.16	0.16	0.16	0.00	1.00
-30	0.16	0.16	0.16	0.16	0.00	1.00
-40	0.16	0.16	0.16	0.16	0.00	1.00
-50	0.16	0.16	0.16	0.16	0.00	1.00
-60	0.16	0.16	0.16	0.16	0.00	1.00
-70	0.16	0.16	0.16	0.16	0.00	1.00
-80	0.16	0.16	0.16	0.16	0.00	1.00
-90	0.16	0.16	0.16	0.16	0.00	1.00

University of Ghana http://ugspace.ug.edu.gh

APPENDIX H

CORRECTION FACTORS FOR BARE FILM

Table H1: Field size correction factors for bare film

ONE SIDE OF SQUARE FIELD (cm)	CORRECTION FACTOR
4	2.670000
6	1.735333
8	1.722364
10	1.000000
12	0.840000
14	0.830000
16	0.711100
18	0.700000
20	0.610000
22	0.552000
24	0.546150

University of Ghana http://ugspace.ug.edu.gh

Table H2: SSD correction factors for bare film

SSD (cm)	CORRECTION FACTOR
75	0.704587
80	0.808074
85	0.836222
90	0.841048
95	0.982216
100	1.000000
110	1.243896
120	2.610000

Table H3: Gantry angle correction factors for bare film

GANTRY ANGLE (˚)	CORRECTION FACTOR
±0	1.000000
±5	0.823963

University of Ghana http://ugspace.ug.edu.gh

±10	1.700000
±15	0.974980
±20	0.973077
±25	1.000000
±30	0.990588
±40	0.973077
±50	0.823077
±60	0.708190
±70	0.554812
±80	0.549655
±90	0.823282

Table H4: Wedge angle correction factors for bare film

One side of square field (cm)	WF 15˚	WF 30˚	WF 45˚	WF 60˚
4	1.246800	1.234340	1.231906	1.232500
10	1.235500	1.262115	1.241220	1.248599

University of Ghana http://ugspace.ug.edu.gh

12	1.235400	1.245520	1.251112	1.253330
14	1.241100	1.259327	1.266667	1.256660
18	1.234500	1.238010	1.271112	1.265440

Table H5: Tray correction factors for bare film

**ONE SIDE OF SQUARE FIELD (cm)**	**CORRECTION FACTOR**
4	1.255540
10	1.232704
12	1.229002
14	0.999321
18	0.974310

University of Ghana http://ugspace.ug.edu.gh

APPENDIX I

PHANTOM STUDIES

ENTRANCE DOSE MEASUREMENTS

Table I1a: Dose comparison to d_{max} for varying Field Size values at reference SSD of 100 cm, open beam geometry and normal beam incidence for bare film (Prescribed dose = 200 cGy)

One side of square field (cm)	Treatment time (minutes)	N.O.D bare film	Measured dose (cGy)	Dose difference	% dose difference
4	1.18	0.02	202.1164	2.1164	1.05821
6	1.15	0.03	204.2215	4.2215	2.11077
8	1.13	0.03	202.6953	2.6953	1.34765
10	1.11	0.05	201.3688	1.3688	0.68440
12	1.09	0.06	204.2526	4.2526	2.12623
14	1.07	0.06	201.8210	1.8210	0.91051
16	1.06	0.07	202.6432	2.6432	1.32158
18	1.04	0.07	199.4800	-0.5200	-0.26001
20	1.03	0.08	199.3812	-0.6188	-0.30939
22	1.02	0.09	203.6063	3.6063	1.80314
24	1.01	0.09	201.4485	1.4485	0.72425

University of Ghana http://ugspace.ug.edu.gh

Table I1b: Dose comparison to d_{max} for random Field Size values at reference SSD of 100 cm, open beam geometry and normal beam incidence for bare film (Prescribed dose = 200 cGy)

One side of square field (cm)	Treatment time (minutes)	N.O.D bare film	Measured dose (cGy)	Dose difference	% dose difference
7	1.13	0.03	200.5000	0.5000	0.25
15	1.06	0.06	201.3900	1.3900	0.70
21	1.02	0.08	201.5700	1.5700	0.79

Table I2: Dose comparison to d_{max} for random gantry angle values at reference field size of 10 cm × 10 cm, open beam geometry and reference SSD of 100 cm for bare film (Prescribed dose = 200 cGy)

Gantry angle (degrees)	Treatment time (minutes)	N.O.D bare film	Measured dose (cGy)	Dose difference	% dose difference
±0	1.11	0.05	201.3688	1.3688	0.6844
±5	1.15	0.06	200.3531	0.3531	0.1765
±10	1.11	0.03	200.0634	0.0634	0.0317
±15	1.12	0.05	196.3305	-3.6695	-1.8340
±20	1.10	0.05	195.9473	-4.0527	-2.0263
±25	1.10	0.05	201.3688	1.3688	0.6844
±30	1.10	0.05	199.4736	-0.5264	-0.2632
±40	1.10	0.05	195.9473	-4.0527	-2.0263
±50	1.10	0.06	200.1376	0.1376	0.0688

±60	1.11	0.07	201.8139	1.8139	0.9069
±70	1.10	0.09	204.6435	4.6435	2.3217
±80	1.15	0.09	202.7413	2.7413	1.3707
±90	2.12	0.06	200.1875	0.1875	0.0937

Table I2a:Dose comparison to d_{max} for random gantry angle values at reference field size of 10 cm × 10 cm, open beam geometry and reference SSD of 100 cm for bare film (Prescribed dose = 200 cGy)

Gantry angle (degrees)	Treatment time (minutes)	N.O.D bare film	Measured dose (cGy)	Dose difference	% dose difference
32	1.19	0.05	196.7500	3.2400	1.6000
62	1.1	0.07	198.7700	1.2300	0.6000

Table I3: Dose comparison to d_{max} for varying SSD values at reference field size of 10 cm × 10 cm, open beam geometry and normal beam incidence for bare film (Prescribed dose = 200 cGy)

SSD (cm)	Treatment time (minutes)	N.O.D bare film	Measured dose (cGy)	Dose difference	% dose difference
75	0.62	0.07	200.7872	0.7872	0.3936
80	0.70	0.06	196.4895	-3.5105	-1.7552
85	0.79	0.06	203.3339	3.3339	1.6669
90	0.89	0.06	204.5074	4.5074	2.2537
95	1.00	0.05	197.7877	-2.2124	-1.1062
100	1.11	0.05	201.3688	1.3688	0.6844

110	1.22	0.04	198.4752	-1.5248	-0.7624
120	1.34	0.02	197.5745	-2.4255	-1.2128

Table I3a: Dose comparison to d_{max} for random SSD values at reference field size of 10 cm × 10 cm, open beam geometry and normal beam incidence for bare film (Prescribed dose = 200 cGy)

One side of square field (cm)	Treatment time (minutes)	Mean N.O.D bare film	Measured dose (cGy)	Dose difference	% dose difference
97	0.96	0.05	195.3277	4.6700	2.3000
100	1.10	0.05	201.3600	1.3600	0.6840
112	1.29	0.03	197.1200	2.8800	1.4000

Table I4: Dose comparison to d_{max} for wedge angle of 15° at reference SSD OF 100 cm, normal beam incidence and varying field size for bare film (prescribed dose = 200 cGy)

One side of square field (cm)	Treatment time (minutes)	N.O.D bare film	Measured dose (cGy)	Dose difference	% dose difference
4	1.54	0.04	198.9470	-1.0530	-0.5265
10	1.45	0.04	197.1398	-2.8602	-1.4301
12	1.42	0.04	197.1259	-2.8740	-1.4370
14	1.40	0.04	198.0290	-1.9709	-0.9855
18	1.37	0.04	196.9839	-3.01608	-1.50804

University of Ghana http://ugspace.ug.edu.gh

Table I5: Dose comparison to d_{max} for wedge angle of 30° at reference SSD OF 100 cm, normal beam incidence and varying field size for bare film (prescribed dose = 200 cGy)

One side of square field (cm)	Treatment time (minutes)	N.O.D bare film	Measured dose (cGy)	Dose difference	% dose difference
4	1.86	0.04	196.9504	-3.0495	-1.5248
10	1.75	0.04	201.3822	1.3822	0.6911
12	1.72	0.04	198.7343	-1.2657	-0.6329
14	1.70	0.04	200.9373	0.9373	0.4686
18	1.65	0.04	197.5360	-2.4640	-1.2320

Table I6: Dose comparison to d_{max} for wedge angle of 45° at reference SSD OF 100 cm, normal beam incidence and varying field size for bare film (Prescribed dose = 200 cGy)

One side of square field (cm)	Treatment time (minutes)	N.O.D bare film	Measured dose (cGy)	Dose difference	% dose difference
4	2.44	0.04	196.5620	-3.4380	-1.7190
10	2.29	0.04	198.0482	-1.9518	-0.9759
12	2.25	0.04	199.6265	-0.3735	-0.1867
14	2.22	0.04	202.1085	2.1085	1.0542
18	2.16	0.04	202.8177	2.8177	1.4089

University of Ghana http://ugspace.ug.edu.gh

Table I7: Dose comparison to d_{max} for wedge angle of 60° at reference SSD OF 100 cm, normal beam incidence and varying field size for bare film (Prescribed dose = 200 cGy)

One side of square field (cm)	Treatment time (minutes)	N.O.D bare film	Measured dose (cGy)	Dose difference	% dose difference
4	4.57	0.04	196.6568	-3.3432	-1.6720
10	4.30	0.04	199.2256	-0.7744	-0.3872
12	4.22	0.04	199.9804	-0.0196	-0.0098
14	4.16	0.04	200.5118	0.5118	0.2559
18	4.06	0.04	201.9127	1.9127	0.9564

Table I8: Dose comparison to d_{max} for tray thickness of 5mm at reference SSD of 100 cm, normal beam incidence and varying field size for bare film (prescribed dose = 200 cGy)

One side of square field (cm)	Treatment time (minutes)	N.O.D bare film	Measured dose (cGy)	Dose difference	% dose difference
4	1.19	0.04	200.3330	0.3330	0.1665
10	1.13	0.04	196.6890	-3.3106	-1.6553
12	1.10	0.04	196.0990	-3.9013	-1.9507
14	1.08	0.05	201.2320	1.2320	0.6160
18	1.05	0.05	196.1960	-3.8043	-1.9022

University of Ghana http://ugspace.ug.edu.gh

APPENDIX J

Phantom studies (Skin dose Estimation)

Table J1: Effect of field size on percentage skin dose

One side of square field (cm)	Correction factors	Absolute Skin dose ($\frac{Dskin}{Dentrance}$)	% Absolute skin dose ($\frac{Dskin}{Dentrance}$ × 100)
4	2.67000	0.08878	8.87800
6	1.73533	0.13661	13.66100
8	1.72236	0.13764	13.76400
10	1.00000	0.23706	23.70600
12	0.84000	0.28221	28.22100
14	0.83000	0.28561	28.56100
16	0.71110	0.33337	33.33700
18	0.70000	0.33866	33.86600
20	0.61000	0.38862	38.86230
22	0.55200	0.42946	42.94565
24	0.54615	0.43405	43.40566

University of Ghana http://ugspace.ug.edu.gh

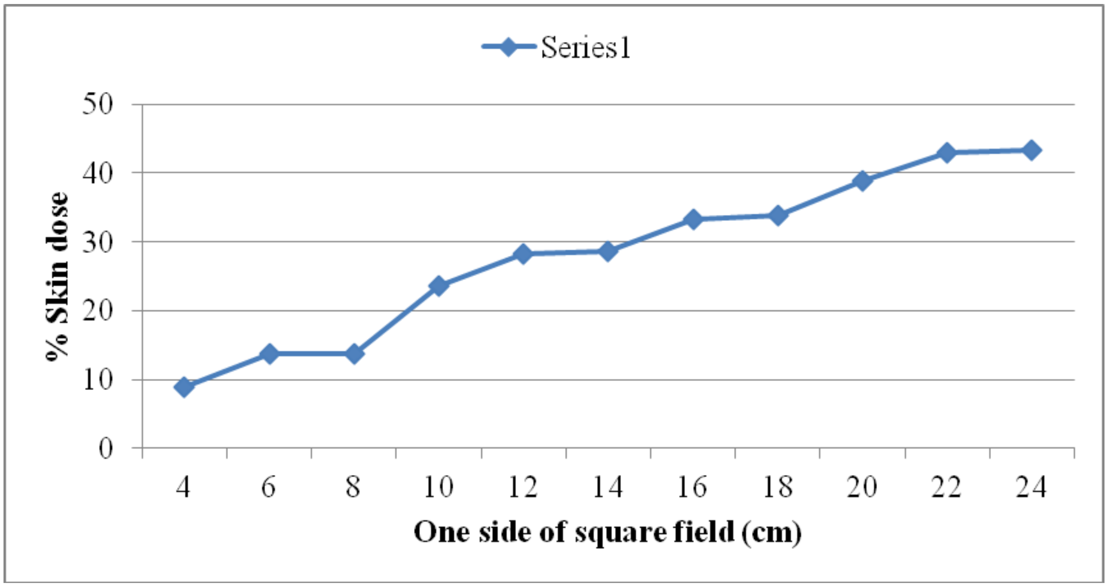

Figure J1: Effect of field size on percentage skin dose

Table J2: Effect of SSD on percentage skin dose

SSD (cm)	Correction factors	Absolute Skin dose ($\frac{Dskin}{Dentrance}$)	% Absolute skin dose ($\frac{Dskin}{Dentrance} \times 100$)
75	0.70458	0.33645	33.64500
80	0.80807	0.29336	29.33600
85	0.83622	0.28348	28.34800
90	0.84105	0.28186	28.18600
95	0.98221	0.24135	24.13500
100	1.00000	0.23706	23.70600
110	1.24389	0.19057	19.05700
120	2.61000	0.09082	9.08200

University of Ghana http://ugspace.ug.edu.gh

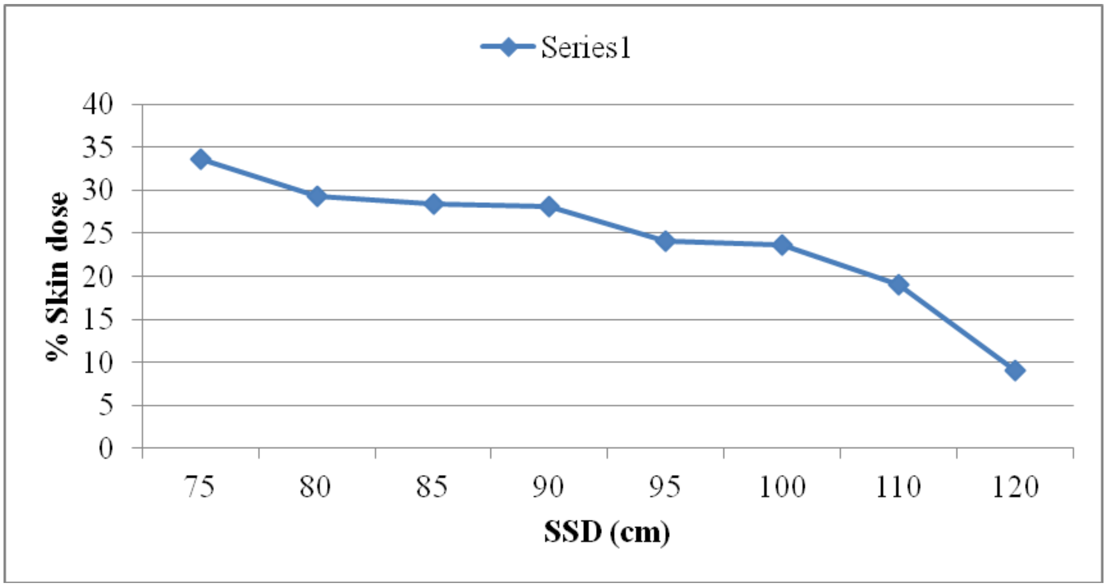

Figure 2J: Effect of SSD on percentage skin dose

Table J3: Effect of gantry angle on percentage skin dose

Gantry angle (˚)	Correction factors	Absolute Skin dose ($\frac{Dskin}{Dentrance}$)	% Absolute skin dose ($\frac{Dskin}{Dentrance} \times 100$)
±0	1.000000	0.2370600	23.70600
±5	0.823960	0.2877000	28.77000
±10	1.700000	0.1394500	13.94500
±15	0.974980	0.2431400	24.31400
±20	0.973080	0.2436200	24.36200
±25	1.000000	0.2370600	23.70600
±30	0.990590	0.2393124	23.93124
±40	0.973080	0.2436190	24.36190
±50	0.823077	0.2880168	28.80168

±60	0.708190	0.3347407	33.47407
±70	0.554812	0.4272799	42.72799
±80	0.549655	0.4312887	43.12887
±90	0.823282	0.2879451	28.79451

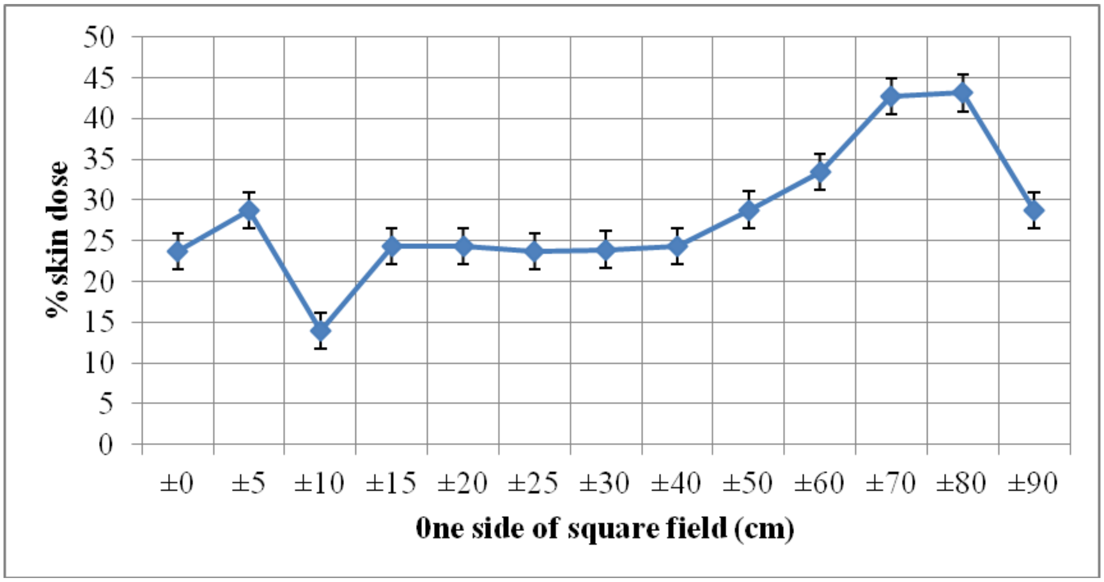

Figure J3: Effect of gantry angle on percentage skin dose

Table J4: Effect of Wedge angle of 15° on percentage skin dose

Field size (cm)	Correction factors	Absolute Skin dose ($\frac{Dskin}{Dentrance}$)	% Absolute skin dose ($\frac{Dskin}{Dentrance} \times 100$)
4	1.246850	0.1901270	19.01270
10	1.235530	0.1918695	19.18695
12	1.235440	0.1918831	19.18831

14	1.24110	0.1910080	19.10080
18	1.23455	0.1920214	19.20214

Table J5: Effect of Wedge angle of 30° on percentage skin dose

Field size (cm)	Correction factors	Absolute Skin dose ($\frac{Dskin}{Dentrance}$)	% Absolute skin dose ($\frac{Dskin}{Dentrance} \times 100$)
4	1.234340	0.1920500	19.20500
10	1.262120	0.1878300	18.78300
12	1.245520	0.1903300	19.03300
14	1.259230	0.1882400	18.82400
18	1.238010	0.19148500	19.14800

Table J6: Effect of Wedge angle of 45° on percentage skin dose

Field size (cm)	Correction factors	Absolute Skin dose ($\frac{Dskin}{Dentrance}$)	% Absolute skin dose ($\frac{Dskin}{Dentrance} \times 100$)
4	1.231910	0.1924300	19.24300
10	1.241202	0.1909900	19.09900
12	1.251110	0.1894800	18.94800
14	1.266670	0.1871500	18.71500
18	1.271110	0.1864900	18.64900

Table J7: Effect of Wedge angle of 60° on percentage skin dose

Field size (cm)	Correction factors	Absolute Skin dose ($\frac{Dskin}{Dentrance}$)	% Absolute skin dose ($\frac{Dskin}{Dentrance} \times 100$)
4	1.232510	0.1923410	19.23410
10	1.248590	0.1898610	18.98610
12	1.253330	0.1891440	18.91440
14	1.256660	0.1886430	18.86430
18	1.265440	0.1873340	18.73340

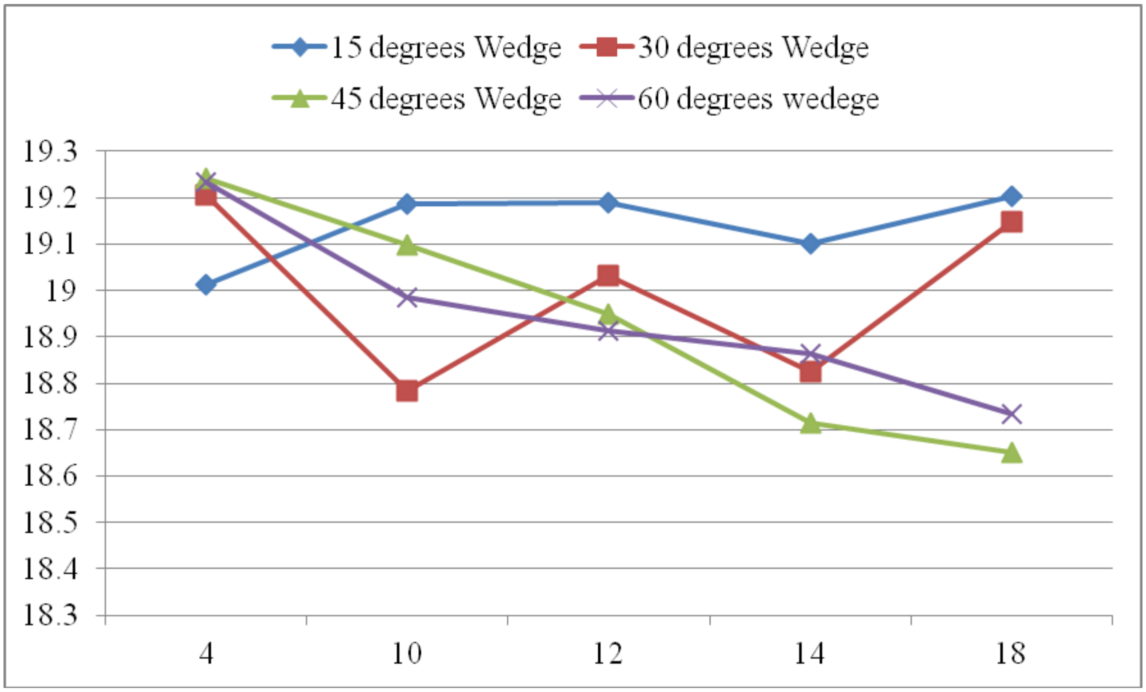

Figure J4: Effect of Wedge angle on percentage skin dose

University of Ghana http://ugspace.ug.edu.gh

Table J8: Effect of tray on percentage skin dose

Field size (cm)	Correction factors	Absolute Skin dose ($\frac{Dskin}{Dentrance}$)	% Absolute skin dose ($\frac{Dskin}{Dentrance} \times 100$)
4	1.255540	0.1888110	18.88110
10	1.232700	0.1923090	19.23090
12	1.229000	0.1928880	19.28880
14	0.999320	0.2372210	23.72210
18	0.974310	0.2433110	24.33110

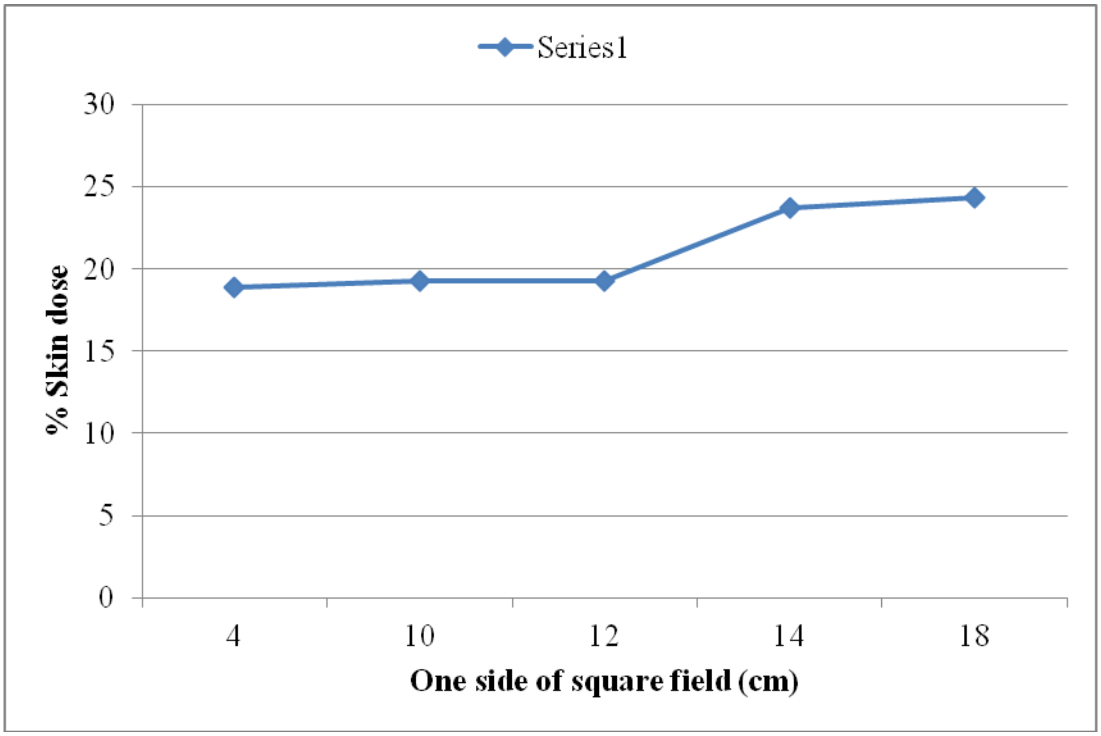

Figure J6: Effect of tray on percentage skin dose

University of Ghana http://ugspace.ug.edu.gh

APPENDIX K

Table K1: Proposed IVD Microsoft Excel Chart

Date	30/07/2014	
Name of Patient	SAMPELE CHARLES	
Treatment Site	PROSTATE	
Prescribed Dose to d_{max} (cGy)	600	
SSD (cm)	100	
SSD correction factor	1	
Equivalent FS	10	
FS corrction factor	1	
GA (degrees)	0	
GA correction factor	1	
Wedge	15	
Wedge factor	1.235527	
Tray (5mm)	YES	
Tray factor	1.232704	
Entrance dose calibration factor	4.134	
Skin dose calibration factor	0.98	
Film Reading in *OD*	0.1	
Film Reading in cGy	99.42157	
Entrance dose (cGy)	=B17*B20*B8*B10*B12*B14*B16	
Dose Deviation (cGy)	25.98241746	
% Dose Deviation	4.33040291	
Skin dose (cGy)	97.4331386	
% Skin dose	15.56483631	

APPENDIX L

Figure L1: Guidelines for carrying out *in vivo* dosimetry (taken from Barcelona for GAF2)

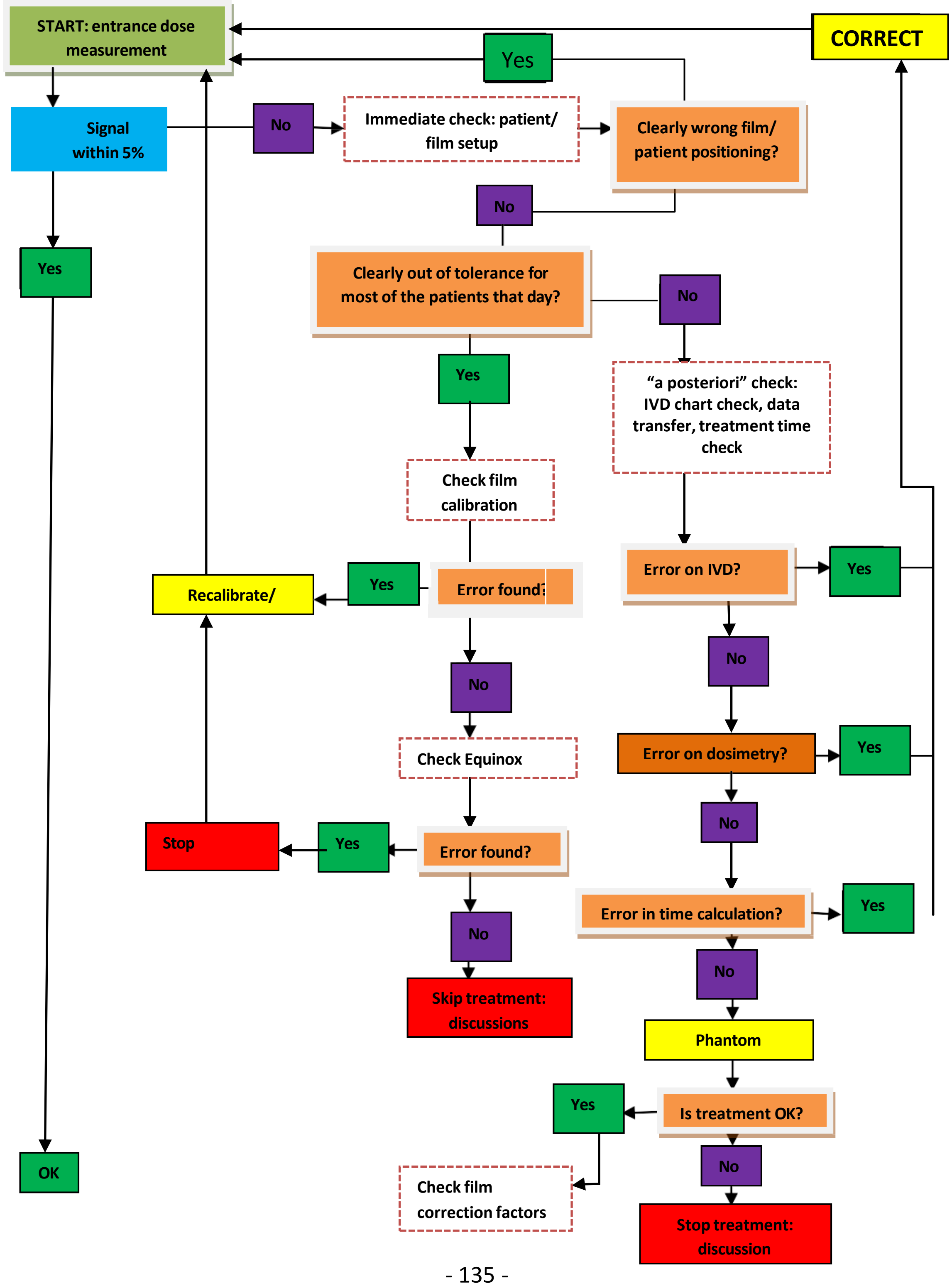

Supplement: S1 Dataset — (PDF) [file pone.0271000.s001.pdf]
