# Supplementary material for: Characterization of GafChromic EBT2 film dose measurements using a tissue-equivalent water phantom for a Theratron® Equinox Cobalt-60 teletherapy machine
Source: PLoS One. 2022 Aug 19;17(8):e0271000. doi: 10.1371/journal.pone.0271000 (PMC9390906; doi:10.1371/journal.pone.0271000)
Supplement: Attachment — Submitted filename: PONE-D-21-35936_authors.pdf [file pone.0271000.s003.pdf]

PLOS ONE

Implementation of in vivo dosimetry using Gafchromic EBT2 film for a Theratron Equinox

PONE-D-21-35936

Suggestions to the authors:

The title should be changed, as it suggests results on the use of the method on real patients will be shown when it is the characterization of the methodology proposed that is being presented.

Lines 107, 151, 498. The average energy of the Co-60 beam is 1.25 MeV (mega electron-volt) and not MV. The unit “MV” is used for megavoltage beams produced by linear accelerators. MeV stands for the energy levels of the nuclear transitions or decay of Co-60 to Ni-60 and emission of the two cascade photons.

In Materials and Methods, please mention the temperature and pressure variations allowed for, or state that correction factors to correct for the response of the ionization chamber due to temperature and pressure differences on measurement day compared to the calibration day, were used.

Lines 238 and 242. A product of correction factors is considered. Which are the correction factors?

Check the figure caption in “Figure 6 – D: Wedge angle”.

Mention that results in Figure 5 were normalized to reference conditions (10x10 cm2; SSD= 100 cm; Gantry angle 0°).

Mention that Figures 6 and 7 were obtained with, or normalized to, the same reference dose.

Line 393, “dose differences” compared to what?

In “Discussion” the first sentence (line 405-406) should be revised. The objective of radiotherapy is to deliver the prescribed dose to the target volume, sparing the surrounding healthy organs and tissues as much as possible. The sentence used is very poor.

In “references” the journal titles are not mentioned.
