# Supplementary material for: Characterization of GafChromic EBT2 film dose measurements using a tissue-equivalent water phantom for a Theratron® Equinox Cobalt-60 teletherapy machine
Source: PLoS One. 2022 Aug 19;17(8):e0271000. doi: 10.1371/journal.pone.0271000 (PMC9390906; doi:10.1371/journal.pone.0271000)
Supplement: Attachment — Submitted filename: Conclusion.doc [file pone.0271000.s002.doc]

1.The implementation of “in vivo” dosimetry method presupposes its clinical application, or demonstration of examples of its clinical application. Meanwhile, based on the data given in the manuscript, there are no examples of clinical application or description of the irradiation conditions of real patients. Therefore, the title of the article does not correspond to its content.

2.Moreover, in the case of clinical examples, information from the authors is required on the compliance with ethical standards when performing research involves humans. This is missing from the manuscript.

2.In addition, there are a number technical errors in the manuscript. The numbering of equations in the paper is violated, for example, after the equation with the number 6 the equation with the number 5 is indicated (?).

Some terms included in the equations do not have an explanation. For example, definition of the term OD1 is not given in equation 1, the term ΠCF is indicated in Equation 4 as "product of correction factors used during the clinical applications", but this term is not clarified in relation to clinical applications.

3.Moreover, estimates of uncertainties are not indicated for the quantitative values given between equations 5 and 6.

Conclusion:

As presented, the paper cannot be recommended for publication in the Journal.
