# Supplementary material for: Characterization of GafChromic EBT2 film dose measurements using a tissue-equivalent water phantom for a Theratron® Equinox Cobalt-60 teletherapy machine
Source: PLoS One. 2022 Aug 19;17(8):e0271000. doi: 10.1371/journal.pone.0271000 (PMC9390906; doi:10.1371/journal.pone.0271000)
Supplement: Attachment — Submitted filename: Response to Reviewers.docx [file pone.0271000.s004.docx]

**RESPONSE TO REVIEWERS**

***REVIEWER 1***

1. The implementation of “in vivo” dosimetry method presupposes its clinical application, or demonstration of examples of its clinical application. Meanwhile, based on the data given in the manuscript, there are no examples of clinical application or description of the irradiation conditions of real patients. Therefore, the title of the article does not correspond to its content.

**Response:** Thank you for the comment. Yes, you are right! The title of the article does not correspond to its content. The title of the research has been changed to “Characterization of GafChromic EBT2 film dose measurements using a tissue-equivalent water phantom for a Theratron® Equinox Cobalt-60 Teletherapy Machine”. This is indicated on line 1-3.

2. Moreover, in the case of clinical examples, information from the authors is required on the compliance with ethical standards when performing research involves humans. This is missing from the manuscript.

**Response:** Thank you for this feedback. The title has been modified since this study did not involve humans. Thus, no documentation on compliance with ethical standards is included in this modified manuscript.

3. In addition, there are a number technical errors in the manuscript. The numbering of equations in the paper is violated, for example, after the equation with the number 6 the equation with the number 5 is indicated (?).

**Response:** Thank you. The numbering of equations has been looked at and order restored. The equation with the number 5 now precedes the equation with the number 6 and equation 7 and 8 is also corrected. This is shown at line 265, 267, 282, 313 respectively.

4. Some terms included in the equations do not have an explanation. For example, definition of the term OD1 is not given in equation 1, the term ΠCF is indicated in Equation 4 as "product of correction factors used during the clinical applications", but this term is not clarified in relation to clinical applications.

**Response:** The term OD1 has already been defined in line 142, however, this has been redefined in line 174 so that it is easy to follow. Since no clinical applications were conducted in this study, ΠCF has now been indicated as "product of correction factors used during the dose estimations; and illustrated on line 246-247".

5. Moreover, estimates of uncertainties are not indicated for the quantitative values given between equations 5 and 6.

**Response:** Thank you for this feedback. Estimates of uncertainties have been included and their effect on the results assessed. This is shown at line 273-275.

***REVIEWER 2***

1. The title should be changed, as it suggests results on the use of the method on real patients will be shown when it is the characterization of the methodology proposed that is being presented.

**Response:** Thank you for the comment. Yes, you are right! The title of the article does not correspond to its content. The title of the research has been changed to “Characterization of GafChromic EBT2 film dose measurements using a tissue-equivalent water phantom for a Theratron® Equinox Cobalt-60 Teletherapy Machine”. This is indicated on line 1-3.

2. Lines 107, 151, 498. The average energy of the Co-60 beam is 1.25 MeV (mega electron-volt) and not MV. The unit “MV” is used for megavoltage beams produced by linear accelerators. MeV stands for the energy levels of the nuclear transitions or decay of Co-60 to Ni-60 and emission of the two cascade photons.

**Response:** The unit of the average energy of Co-60 beam has been changed to 1.25 MeV throughout the manuscript.

3. In Materials and Methods, please mention the temperature and pressure variations allowed for, or state that correction factors to correct for the response of the ionization chamber due to temperature and pressure differences on measurement day compared to the calibration day, were used.

**Response: “**Correction factors to correct for the response of the ionization chamber due to temperature and pressure differences on measurement day compared to the calibration day” text has been included to account for possible variations in the response of the ionization chamber.

4. Lines 238 and 242. A product of correction factors is considered. Which are the correction factors?

**Response:** The correction factors considered have been listed at those lines and they are correction factors for field size, SSD, gantry angle and wedge angle. This is shown on line 246 and 247.

5. Check the figure caption in “Figure 6 – D: Wedge angle”.

**Response:** Thank you for the comment. The figure caption has been modified and reads “Figure 6: Effects of irradiation parameters on skin dose deposition. A: Effect of Field size; B: Effect of SSD; C: Effect of Gantry angle; D: Effect of Wedge angle”.

6. Mention that results in Figure 5 were normalized to reference conditions (10x10 cm^2^; SSD= 100 cm; Gantry angle 0^o^).

**Response:** Thank you. In the results section where Figure 5 is located, statements have been added to emphasize that results in Figure 5 were normalized to reference conditions (10x10 cm^2^; SSD= 100 cm; Gantry angle 0^o^).

7. Mention that Figures 6 and 7 were obtained with, or normalized to, the same reference dose.

**Response:** Thank you for this feedback. Statements have been included to emphasize that Figures 6 and 7 were obtained with the same reference dose. This is indicated on line 386-387 and line 398-399 for Figure 6 and line 406-407 and line 411-412 for Figure 7.

8. Line 393, “dose differences” compared to what?

**Response:** Thank you. The dose differences were determined by comparing dose estimates using the films and the prescribed dose expected to be delivered to dmax (that is 200 cGy). This has been indicated on line 406-408.

9. In “Discussion” the first sentence (line 405-406) should be revised. The objective of radiotherapy is to deliver the prescribed dose to the target volume, sparing the surrounding healthy organs and tissues as much as possible. The sentence used is very poor.

**Response:** The first sentence of the “Discussion” Section has been revised and now reads “Radiation therapy involves the delivery of a prescribed dose to the target volume, sparing the surrounding healthy organs and tissues as much as possible”. This is indicated on line 420-421.

10. In “references” the journal titles are not mentioned.

**Response:** Thank you. The journal titles have now been included in the “references”.
